# Knowledge, attitude, and practice of embryo transfer among women who underwent *in vitro* fertilization-embryo transfer

**DOI:** 10.3389/fcell.2024.1405250

**Published:** 2024-08-07

**Authors:** Yangying Xu, Cuifang Hao, Huimin Zhang, Yingxue Liu, Wei Xue

**Affiliations:** Reproductive Medicine Center of Qingdao Women and Children’s Hospital Affiliated with Qingdao University, Qingdao, China

**Keywords:** knowledge, attitude, practice, embryo transfer, *in vitro* fertilization

## Abstract

**Introduction:**

The infertile patient’s knowledge, attitude, and practice (KAP) toward embryo transfer may affect treatment outcomes and the mental health of women who underwent *in vitro* fertilization-embryo transfer (IVF-ET). This study aimed to investigate the KAP of embryo transfer among women who underwent IVF-ET.

**Methods:**

This cross-sectional study was conducted on women who underwent IVF-ET at our Hospital between May 2023 and November 2023, using a self-designed questionnaire.

**Results:**

A total of 614 valid questionnaires were finally included. The mean KAP scores were 19.46 ± 5.06 (possible range: 0 28), 39.41 ± 5.20 (possible range: 12–60), and 48.02 ± 6.75 (possible range: 0–60), respectively. The structural equation model demonstrated that knowledge has a direct effect on attitude (β = 0.27, *p* < 0.001) and attitude has a direct effect on practice (β = 0.55, *p* < 0.001) and anxiety (β = 0.59, *p* < 0.001). Moreover, multivariable linear regression analysis showed that anxiety score [coefficient = 0.09, 95% confidence interval (CI): 0.03–0.16, *p* = 0.003], BMI (coefficient = 0.09, 95%CI: 0.03–0.16, *p* = 0.003), education (coefficient = 5.65–6.17, 95%CI: 1.09–10.7, *p* < 0.05), monthly per capita income (coefficient = 1.20–1.96, 95% CI: 0.21–3.07, *p* = 0.05), reasons for IVF (coefficient = −1.33–1.19, 95% CI: −2.49–0.09, *p* < 0.05), and more than 5 years of infertility (coefficient = −1.12, 95% CI: −2.11–0.13, *p* = 0.026) were independently associated with sufficient knowledge. Knowledge (coefficient = 0.19, 95% CI: 0.12–0.26, *p* < 0.001), anxiety (coefficient = 0.39, 95% CI: 0.34–0.45, *p* < 0.001), monthly per capita household income >10,000 (coefficient = 1.52, 95% CI: 0.61–2.43, *p* < 0.001), and three or more cycles of embryo transfer (coefficient = −2.69, 95% CI: −3.94–1.43, *p* < 0.001) were independently associated with active attitude. Furthermore, attitude (coefficient = 0.21, 95% CI: 0.11–0.30, *p* < 0.001) and anxiety (coefficient = 0.57, 95% CI: 0.49–0.65, *p* < 0.001) were independently associated with proactive practice.

**Discussion:**

Women who underwent IVF-ET had inadequate knowledge and negative attitudes but proactive practice toward embryo transfer, which were affected by anxiety, income, and reasons for IVF. It is necessary to strengthen the continuous improvement of patient education to improve the management of embryo transfer.

## Introduction

Infertility is defined as 12 months of unprotected intercourse without successful conception. There are more than 186 million people worldwide who suffer from infertility, accounting for 8%–12% of couples of reproductive age (Inhorn and Patrizio, 2015). *In vitro* fertilization (IVF)-embryo transfer (ET), a widely adopted assisted reproductive technology (ART), solves the problem of infertility to a certain extent and offers hope for infertile patients (De Geyter, 2019). ET is the most critical and important link in the process of IVF-ET treatment. Medical staff have also done a lot of propaganda and education work before IVF-ET, but patients are still full of expectation and fear and accompanied by great psychological pressure. Some patients also have abdominal pain, lower back pain, insomnia, and other physical symptoms after this very minimally invasive surgery, which may affect the success rate of patients to a certain extent. The psychological wellbeing of patients undergoing IVF-ET procedures varies significantly, which may be influenced by the knowledge, attitude, and practice (KAP) of ET among infertile patients (Yang et al., 2021). Therefore, it is crucial to understand the KAP level of ET among infertile individuals undergoing IVF-ET procedures, as this will assist in optimizing ART treatment plans and improving the success rates of ART procedures while also considering the overall health and wellbeing of the patient.

KAP study, as a valuable survey methodology, has been widely applied in gaining insight into patients’ perceptions, emotions, and behaviors regarding specific medical topics (Moon and Hyun, 2019; Pandey et al., 2020; Tahani and Manesh, 2021). Past research has predominantly focused on the fields of medicine and biology, with a focus on technological advancements and healthcare professionals’ experiences (Justin et al., 2022). However, as the patient’s level of KAP plays a crucial role in the outcomes of the treatment process, it is crucial to take into account this aspect. Unfortunately, previous studies have primarily focused on knowledge and attitude regarding ART, and most of these surveys are outdated (more than 20 years old) (Braverman et al., 1998), limited in size (Anaman-Torgbor et al., 2021), targeted to specific populations (Brandão et al., 2023), or conducted in other countries (Szalma and Bitó, 2021). Currently, there is a lack of in-depth understanding of KAP about ET among patients undergoing IVF-ET, which poses a significant challenge for precise adjustment of treatment regimens and establishment of patient support systems. Therefore, this study aimed to investigate the KAP of ET among women who underwent IVF-ET.

## Methods

### Study design and subjects

This cross-sectional study was conducted between May 2023 and November 2023 in Qingdao Women and Children’s Hospital and enrolled infertility patients who underwent IVF-ET. Inclusion criteria: 1) female patients with infertility or sterility diagnosed in both or one of the spouses; 2) patients who underwent ET; 3) body mass index (BMI) ranging from 17 to 36 kg/m^2^; 4) consent to participate in the study. Exclusion criteria: 1) unavailability of blood HCG 14 days after ET; 2) patients who were unable to cooperate with the questionnaire. The study was approved by the Institutional Ethics Committee of Qingdao Women and Children’s Hospital of Qingdao University [QFELL-YJ-2023-105]. All participants were informed about the study protocol and provided informed consent to participate in the study.

### Questionnaire introduction

The questionnaire was designed with reference to ET guidelines and literature (Penzias et al., 2017; Practice Committee of the American Society for Reproductive Medicine. Electronic address: ASRM@asrm.orgPractice Committee of the American Society for Reproductive Medicine, 2017; Hairston et al., 2020; Cheung et al., 2019), designed and modified with reference to comments made by five reproductive experts, and administered on a small scale (39 copies) with a reliability of 0.888.

The final questionnaire was in Chinese and contained four dimensions: demographic information (age, employment, height, weight, 1 year of residence and work environment, education, income, history of spontaneous abortion, reason for IVF-ET, type of infertility, number of years of infertility, cycles of ET, and the underlying disease), the knowledge dimension, the attitude dimension, and the practice dimension. The knowledge dimension consisted of 14 questions, with a score of 2 for very well known, 1 for somewhat known, and 0 for not known, and a score range of 0–26, with question 14 being descriptive only. The attitude dimension consisted of 12 questions using a five-point Likert scale with questions 1, 6, 10, and 12 ranging from strongly agree (Moon and Hyun, 2019) to strongly disagree (Inhorn and Patrizio, 2015) and *vice versa* for the rest of the questions, with a score range of 12–60. The practice dimension consisted of 12 questions, ranging from always (5 points) to never (1 point), with scores ranging from 12–60. A total score of>70% for each dimension was defined as adequate knowledge, positive attitude, and proactive practice (Lee and Suryohusodo, 2022). Anxiety was assessed by the patient’s anxiety using questions 1–19 of the Anxiety Mood Scale with a total score of 19–76.

### Questionnaire distribution and quality control

Questionnaires were administered to the study participants by paper questionnaire and the Wenjuanxing e-questionnaire platform (Wenjuanxing Tech Co., Ltd., Changsha, China) in the form of a QR code. The online questionnaire was distributed via Questionnaire Star (https://www.wjx.cn) to participants. Participants could scan the QR code using WeChat or follow the provided link to access and complete the questionnaire. To maintain data quality and ensure comprehensive responses, a one-submission-per-IP address restriction was enforced, and all questionnaire items were mandatory. Participants were assured of anonymity during the survey process. The research team, comprising three doctors trained as research assistants responsible for questionnaire promotion and distribution, meticulously reviewed all submissions for completeness, internal consistency, and logical coherence. Investigators were trained to grasp the problem’s meaning and the investigation process, enhancing data accuracy and consistency. Questionnaires containing logical errors, incomplete answers, or uniform responses across all items were categorized as invalid.

### Statistical analysis

The sample size was determined to be 5–10 times the number of questionnaire items (Ping et al., 2010), which, in this case, was 57 independent variables. Consequently, the minimum required sample size was calculated to be 285 participants. To account for a potential 20% invalidity rate among survey questionnaires, a minimum of 357 participants were needed to ensure adequate valid responses.

The statistical software was SPSS 26.0 (IBM Inc., Armonk, NY, United States) and STATA 14.0 (StataCorp, Texas, United States). Continuous data with a normal distribution were described as means ± standard deviations (SD) and analyzed using Student’s t-test and ANOVA. Categorical indicators were described using frequencies (percentages). Sperman’s correlation analysis was used to analyze the correlation between knowledge, attitude, and practice scores. Since better knowledge implies a better attitude, which leads to better practice, three stratified stepwise linear regressions were conducted. The first linear regression had knowledge as the dependent variable (DV) and sociodemographic variables and anxiety as independent variables (IVs). The second linear regression included attitude as the DV and sociodemographic variables and anxiety and knowledge as the IVs. The third regression included practice as the DV and sociodemographic variables, anxiety, knowledge, and attitude as the IVs. The IVs included in these regressions were those that had a *p* < 0.5 in bivariable analyses. Because better knowledge and attitude may reduce anxiety, anxiety scores were used as the DV, sociodemographic variables, and knowledge and attitude as IVs for linear regression analysis. Structural equation modeling (SEM) was used to test the following hypotheses: 1) Knowledge has an effect on attitude; 2) Knowledge has an effect on practice and anxiety; and 3) Attitude has an effect on practice and anxiety. Two-sided *p*-values <0.05 were considered statistically significant.

## Results

### Demographic information

A total of 700 women were enrolled. The individuals were excluded for the following reasons: 3 cases for misunderstanding the “title” in the data, 21 cases for short answer time, 14 cases for out-of-range BMI, 9 cases for choosing other options in answering “4. Your living and working environment in the past year (multiple choices are allowed),” 10 cases for choosing other options in answering “12. The underlying diseases you currently suffer from (multiple choices allowed),” 12 cases for repeated answers in answering question 6 of the knowledge dimension, 2 cases for missing answer of question 29 in the practice dimension, and 15 cases for missing answer of the pregnancy outcome question. Finally, 614 valid questionnaires remained, with an effective response rate of 87.71%. Among the respondents, most were aged 31–40 years, and most had full-time work situations (>60%), with an average BMI of 23.44 ± 3.28. More than 70% of respondents had a college/undergraduate degree or higher and had a per capita monthly household income of more than 5,000. The anxiety score was 61.97 ± 6.04 (possible range: 19–76). Moreover, more than 70% of respondents had no history of spontaneous abortion and less than 5 years of infertility. In this survey, the two most common causes of IVF were tubal infertility (35.5%) and oligozoospermia, weak or abnormal sperm in the male partner (19.71%), and the most common cycles of ET were 1 (63.03%) and 2 (20.36%). The anxiety score was 61.97 ± 6.04 (possible range: 19–76). (Table 1).

**TABLE 1:** Demographic characteristics and KAP scores.

*N* = 614	N (%)	Knowledge	*p*	Attitude	*p*	Practice	*p*
Total Score		19.46 ± 5.06		39.41 ± 5.20		48.02 ± 6.75	
Age			0.007		0.549		0.084
20–30 years old	134 (21.82)	20.08 ± 5.07		39.38 ± 5.20		47.04 ± 6.41	
31–35 years old	274 (44.63)	19.77 ± 4.89		39.64 ± 5.04		47.96 ± 6.89	
36–40 years old	147 (23.94)	19.02 ± 5.27		39.36 ± 5.43		48.52 ± 7.10	
≥40 years old	59 (9.61)	17.66 ± 4.84		38.55 ± 5.31		49.25 ± 5.56	
Employment			0.080		0.027		0.470
Full-time job	371 (60.42)	19.83 ± 4.73		39.88 ± 5.53		48.09 ± 6.87	
Part-time/Housewife	114 (18.57)	5.513058		4.472075		47.38 ± 6.63	
Freelance	129 (21.01)	19.21 ± 5.44		38.91 ± 4.62		48.36 ± 6.46	
BMI, kg/m^2^	23.44 ± 3.28						
Living and working environment in the last year			0.164		0.086		0.019
No pollution	555 (90.39)	19.55 ± 5.02		39.50 ± 5.15		48.23 ± 6.67	
Yes (noise/decoration/chemical pollution)	59 (9.61)	18.55 ± 5.27		38.57 ± 5.55		46 ± 7.12	
Education			0.002		0.003		0.559
Primary school and below	5 (0.81)	12 ± 3.31		37.8 ± 5.01		50.4 ± 9.55	
Junior high school	74 (12.05)	18.20 ± 5.44		37.83 ± 4.20		48.63 ± 6.83	
High school/secondary school	93 (15.15)	18.90 ± 5.36		38.63 ± 4.40		48.03 ± 7.00	
College/Undergraduate	369 (60.1)	19.79 ± 4.88		39.65 ± 5.29		47.75 ± 6.68	
Master and above	73 (11.89)	20.23 ± 4.59		40.90 ± 6.04		48.56 ± 6.50	
Monthly per capita income			<0.001		<0.001		0.293
<5,000	174 (28.34)	18.12 ± 5.40		37.77 ± 5.13		47.45 ± 7.28	
5,000–10,000	253 (41.21)	19.66 ± 4.94		39.52 ± 4.83		47.97 ± 6.45	
>10,000	187 (30.46)	20.42 ± 4.61		40.79 ± 5.31		48.61 ± 6.58	
History of spontaneous abortion			0.027		0.496		0.517
1	109 (17.75)	19.84 ± 4.89		39.51 ± 5.32		48.04 ± 6.28	
2 or more	63 (10.26)	20.85 ± 4.98		38.71 ± 4.72		47.15 ± 6.80	
None	442 (71.99)	19.16 ± 5.07		39.48 ± 5.23		48.14 ± 6.85	
Reasons for IVF			0.007		0.735		0.541
Tubal infertility	218 (35.5)	19.81 ± 4.95		39.74 ± 5.06		47.62 ± 6.93	
Oligospermia, weak, or abnormal spermatozoa in the male partner	121 (19.71)	18.57 ± 5.33		39.33 ± 4.98		48.49 ± 6.28	
Endometriosis	28 (4.56)	21.25 ± 4.75		39.85 ± 5.62		48.53 ± 7.74	
unexplained infertility	77 (12.54)	18.24 ± 4.72		38.41 ± 4.67		48.14 ± 6.83	
polycystic ovary syndrome	63 (10.26)	20.82 ± 4.27		39.42 ± 5.90		46.95 ± 6.97	
Ovarian hypoplasia	33 (5.37)	18.78 ± 5.29		38.90 ± 4.93		49.18 ± 5.35	
Others	74 (12.05)	19.59 ± 5.39		39.64 ± 5.77		48.48 ± 6.83	
Types of infertility			0.681		0.735		0.377
Primary infertility (never been pregnant before)	345 (56.19)	19.39 ± 4.97		39.43 ± 5.13		48.20 ± 6.72	
Secondary infertility (previous pregnancy)	269 (43.81)	19.53 ± 5.16		39.39 ± 5.28		47.79 ± 6.77	
Years of infertility			0.003		0.107		0.995
Less than 3 years	299 (48.70)	20.06 ± 4.81		39.86 ± 5.26		47.99 ± 6.93	
3–5 years	145 (23.62)	19.53 ± 4.99		39.04 ± 4.98		48.19 ± 6.22	
More than 5 years	170 (27.69)	18.33 ± 5.36		38.92 ± 5.21		47.92 ± 6.86	
Cycle of the embryo transfer			0.164		0.002		0.295
1	387 (63.03)	19.15 ± 5.12		39.88 ± 5.26		48.36 ± 6.77	
2	125 (20.36)	19.92 ± 4.74		39.39 ± 4.69		47.67 ± 7.03	
3	51 (8.31)	19.58 ± 4.93		38 ± 5.09		47.09 ± 6.25	
3 or more	51 (8.31)	20.52 ± 5.30		37.27 ± 5.24		47.19 ± 6.27	
Underlying disease			0.460		0.276		0.024
None	513 (83.55)	19.40 ± 5.01		39.48 ± 5.08		48.28 ± 6.68	
Yes	101 (16.45)	19.73 ± 5.30		39.02 ± 5.72		46.71 ± 6.92	
Anxiety score	61.97 ± 6.04						

### KAP scores

The mean scores for knowledge, attitude, and practice were 19.46 ± 5.06 (possible range: 0–28), 39.41 ± 5.20 (possible range: 12–60), and 48.02 ± 6.75 (possible range: 0–60), respectively. The knowledge scores varied from infertile patients with different ages (*p* = 0.007), education (*p* = 0.002), income level (*p* < 0.001), spontaneous abortion history (*p* = 0.027), and the reasons for IVF-ET (*p* = 0.007). Moreover, infertile patients with different work situations (*p* = 0.027), education (*p* = 0.003), income level (*p* < 0.001), and ET cycle (*p* = 0.002) were more likely to differ in their attitude scores. Regarding the practice scores, there were significant differences among infertile patients in living and working environments in the last year (*p* = 0.019) and the underlying diseases (*p* = 0.024) (Table 1).

### Distribution of KAP dimensions

The question with the highest “Very well-known” rate was “After entering the treatment cycle, you need to follow the doctor’s instructions to take medication, not to take/stop taking medication on your own, and the importance of regular follow-ups (K12),” with a “Very well known” rate of 88.76%. The question with the lowest “Very well known” rate was “Treatment process during embryo transfer (K13)”, with a “Very well known” rate of 14.01%. Additionally, the questions with the lower “Very well known” rate also included “The process of embryo transfer (K2),” “The reason for holding your urine before the transfer (K3),” and “Dietary precautions taken after the transfer (K6),” with “Very well known” rate of 40.55%, 44.14%, and 44.46%, respectively (Supplementary Table S1).

Supplementary Table S2 presents the distribution of attitude dimension. More than 80% of the respondents strongly agreed or agreed with the viewpoints of “Concerned about the success of the implantation and delivery of your baby during the IVF-EMT procedure (A4),” “Family will understand what you are going through (A6),” “It is important to cooperate with the doctor’s treatment plan and to communicate with the medical staff in a timely manner for the embryo transfer treatment (A10),” and “Feel relaxed by the warm words of doctors and nurses (A12)”. However, more than half of the respondents strongly disagreed or disagreed with the opinions of “There is a difference between embryo transfer and babies born from natural pregnancies (A8)” and “Not trust the surgeon who performed the embryo transfer (A11)”.

In Supplementary Table S3, the distribution of the practice dimension about post-ET can be observed. After ET, more than 70% of the respondents rarely or never had sex-related dreams, pain in lower limbs, cold sweats and insomnia, cold sweats, and fright, cold in lower abdomen and limbs, worried that the doctor would transfer the embryos outside the womb, and felt discomfort, cramps, and soreness in lower limbs after the ET. Notably, over 20% of the respondents always or often worried about the outcome of pregnancy and about having an ectopic pregnancy or miscarriage after the Et. In addition, 65.31% of the respondents had pregnancy outcomes greater than 100 mIU/mL, indicating that these individuals had successful pregnancies after ET.

### Relationship between KAP dimensions

The correlation analysis between KAP dimensions is displayed in Table 2. Knowledge was positively corrected with attitude (r = 0.247, *p* < 0.001), anxiety (r = 0.140, *p* = 0.001), and practice (r = 0.151, *p* < 0.001). Attitude was positively corrected with anxiety (r = 0.509, *p* < 0.001) and practice (r = 0.463, *p* < 0.001). Also, there was a positive correlation between anxiety and practice (r = 0.598, *p* < 0.001).

**TABLE 2:** Correlation analysis.

	Knowledge	Attitude	Anxiety	Practice
Knowledge	1			
Attitude	0.247 (*p* < 0.001)	1		
Anxiety	0.140(*p* = 0.001)	0.509(*p* < 0.001)	1	
Practice	0.151 (*p* < 0.001)	0.463 (*p* < 0.001)	0.598 (*p* < 0.001)	1

**FIGURE 1:**
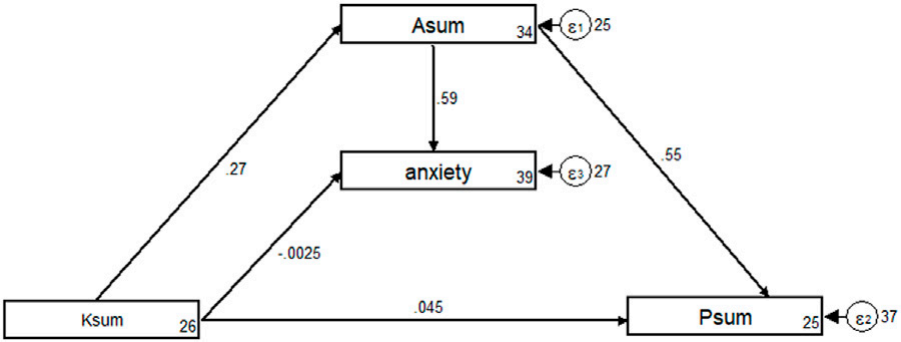
Structural equation modeling analysis.

The fit of the SEM model yielded good indices demonstrating an acceptable model fit (Supplementary Table S4), and the results of the SEM showed knowledge significantly affected attitude (β = 0.27, *p* < 0.001). Additionally, attitude had a significant positive effect on practice (β = 0.55, *p* < 0.001) and anxiety (β = 0.59, *p* < 0.001). However, knowledge had no direct effect on practice and anxiety (*p* > 0.05) (Table 3; Figure 1).

**TABLE 3:** SEM results.

		Estimate	*p*>|z|
Asum < -			
	Ksum	0.27	<0.001
Psum < -			
	Asum	0.55	<0.001
	Ksum	0.04	0.374
anxiety < -			
	Asum	0.59	<0.001
	Ksum	−0.002	0.953

Moreover, multivariable linear regression analysis showed that anxiety score (coefficient = 0.09, 95% confidence interval (CI): 0.03–0.16, *p* = 0.003), BMI (coefficient = 0.09, 95%CI: 0.03–0.16, *p* = 0.003), education [coefficient = 5.65, 95%CI: 1.09–10.2, *p* = 0.013; high school/secondary school, coefficient = 6.07, 95% CI: 1.55–10.6, *p* = 0.009; college/undergraduate, coefficient = 6.17, 95% CI: 1.67–10.6, *p* = 0.007; master and above, coefficient = 6.03, 95% CI: 1.35–10.7, *p* = 0.012], monthly per capita income (5,000–10000, coefficient = 1.20, 95% CI: 0.21–2.20, *p* = 0.018; >10,000, coefficient = 1.96, 95% CI: 0.84–3.07, *p* = 0.001), performing IVF for oligospermia, weak or abnormal spermatozoa in the male partner (coefficient = −1.33, 95% CI: −2.44–0.2, *p* = 0.017), and more than 5 years of infertility (coefficient = −1.12, 95% CI: −2.11–0.13, *p* = 0.026) were independently associated with sufficient knowledge (Table 4).

**TABLE 4:** Univariable and multivariable analysis for knowledge.

Knowledge	Univariable analysis		Multivariable analysis	
	Coefficient (95%CI)	*p*	Coefficient (95%CI)	*p*
			***R*** ^**2**^ =0.0831*	
			**F = 3.78 (*p*<0.001)**	
Anxiety score	0.11 (0.04,0.17)	**0.001**	0.09 (0.03,0.16)	**0.003**
Age				
20–30 years old				
31–35 years old	−0.31 (-1.35,0.72)	0.551	−0.46 (-1.51,0.58)	0.388
36–40 years old	−1.06 (-2.24,0.10)	0.075	−0.87 (-2.09,0.34)	0.159
≥40 years old	−2.42 (-3.96,-0.88)	**0.002**	−1.51 (-3.11,0.09)	0.065
Employment				
Full-time job				
Part-time/Housewife	−1.33 (-2.39,-0.27)	**0.013**	−0.16 (-1.31,0.99)	0.782
Freelance	−0.62 (-1.63,0.39)	0.228	−0.29 (-1.39,0.81)	0.604
BMI, kg/m^2^	−0.09 (-0.21,0.02)	0.134	0.09 (0.03,0.16)	**0.003**
Living and working environment in the last year (multiple choices allowed)				
No pollution				
Yes (noise/decoration/chemical pollution)	−0.99 (-2.35,0.36)	0.151		
Education				
Primary school and below				
Junior high school	6.20 (1.67,10.7)	**0.007**	5.76 (1.22,10.2)	0.013
High school/secondary school	6.90 (2.40,11.4)	**0.003**	6.07 (1.55,10.6)	0.009
College/Undergraduate	7.79 (3.38,12.2)	**0.001**	6.17 (1.67,10.6)	0.007
Master and above	8.23 (3.70,12.7)	**<0.001**	6.03 (1.35,10.7)	0.012
Monthly per capita income				
<5,000				
5,000–10,000	1.53 (0.56,2.49)	**0.002**	1.20 (0.21,2.20)	0.018
>10,000	2.30 (1.27,3.33)	**<0.001**	1.96 (0.84,3.07)	0.001
History of spontaneous abortion				
1				
2 or more	1.01 (-0.55,2.57)	0.204		
None	−0.67 (-1.73,0.37)	0.208		
Reasons for IVF				
Tubal infertility				
Oligospermia, weak or abnormal spermatozoa in the male partner	−1.24 (-2.35,-0.12)	**0.029**	−1.33 (-2.42,-0.2)	0.017
Endometriosis	1.43 (-0.53,3.41)	0.153	1.25 (-0.69,3.20)	0.206
unexplained infertility	−1.56 (-2.86,-0.26)	**0.019**	−1.19 (-2.49,0.09)	0.07
polycystic ovary syndrome	1.01 (-0.39,2.41)	0.158	1.26 (-0.12,2.64)	0.074
Ovarian hypoplasia	−1.02 (-2.86,0.812)	0.274	−0.42 (-2.25,1.41)	0.65
Others	−0.21 (-1.54,1.10)	0.747	−0.13 (-1.43,1.15)	0.833
Types of infertility				
Primary infertility (never been pregnant before)				
Secondary infertility (previous pregnancy)	0.14 (-0.66,0.95)	0.73		
Years of infertility				
Less than 3 years				
3–5 years	−0.53 (-1.52,0.46)	0.294	−0.30 (-1.29,0.68)	0.541
More than 5 years	−1.72 (-2.67,-0.78)	**<0.001**	−1.12 (-2.11,-0.13)	0.026
Cycle of the embryo transfer				
1				
2	0.76 (-0.25,1.78)	0.14		
3	0.43 (-1.04,1.91)	0.563		
3 or more	1.37 (-0.10,2.85)	0.068		
Underlying disease				
None				
Yes	0.32 (-0.75,1.40)	0.553		

Bold values indicate that *p* < 0.05.

#### Adj R-squared

Knowledge (coefficient = 0.19, 95% CI: 0.12–0.26, *p* < 0.001), anxiety (coefficient = 0.39, 95% CI: 0.34–0.45, *p* < 0.001), monthly per capita household income >10,000 (coefficient = 1.52, 95% CI: 0.61–2.43, *p* < 0.001), and three or more cycles of ET (coefficient = −2.69, 95% CI: −3.94–1.43, *p* < 0.001) were independently associated with active attitude (Table 5).

**TABLE 5:** Univariable and multivariable analysis for attitude.

Attitude	Univariable analysis		Multivariable analysis	
	Coefficient (95%CI)	*p*	Coefficient (95%CI)	*p*
			***R*** ^**2**^ =0.3307*	
			**F = 34.65 (*p*<0.001)**	
Knowledge score	0.27 (0.18,0.34)	**<0.001**	0.19 (0.12,0.26)	**<0.001**
Anxiety score	0.43 (0.36,0.49)	**<0.001**	0.39 (0.34,0.45)	**<0.001**
Age				
20–30 years old				
31–35 years old	0.26 (-0.81,1.33)	0.633		
36–40 years old	−0.02 (-1.23,1.19)	0.974		
≥40 years old	−0.82 (-2.41,0.77)	0.312		
Employment	0.32 (-0.75,1.40)	0.553		
Full-time job				
Part-time/Housewife	−1.45 (-2.54,-0.37)	**0.009**	−0.32 (-1.23,0.58)	0.485
Freelance	−0.97 (-2.01,0.06)	0.066	−0.72 (-1.57,0.13)	0.099
BMI, kg/m^2^	0.08 (-0.04,0.20)	0.216		
Living and working environment in the last year				
No pollution				
Yes (noise/decoration/chemical pollution)	−0.92 (-2.32,0.47)	0.193		
Education				
Primary school and below				
Junior high school	0.04 (-4.62,4.70)	0.987		
High school/secondary school	0.83 (−3.80,5.47)	0.724		
College/Undergraduate	1.85 (−2.69,6.40)	0.424		
Master and above	3.10 (-1.56,7.77)	0.192		
Monthly per capita income				
<5,000				
5,000–10000	1.745 (0.76,2.72)	**0.001**	0.75 (-0.08,1.58)	0.078
>10,000	3.02 (1.96,4.06)	**<0.001**	1.52 (0.61,2.43)	**0.001**
History of spontaneous abortion				
1				
2 or more	−0.79 (-2.41,0.81)	0.332		
None	−0.02 (-1.11,1.06)	0.964		
Reasons for IVF				
Tubal infertility				
Oligospermia, weak or abnormal spermatozoa in the male partner	−0.41 (-1.57,0.74)	0.48		
Endometriosis	0.11 (-1.94,2.16)	0.917		
unexplained infertility	−1.33 (-2.68,0.02)	0.054		
polycystic ovary syndrome	−0.31 (-1.78,1.14)	0.668		
Ovarian hypoplasia	−0.83 (-2.74,1.06)	0.388		
Others	−0.09 (-1.47,1.27)	0.888		
Types of Infertility				
Primary infertility (never been pregnant before)				
Secondary infertility (previous pregnancy)	−0.04 (-0.87,0.78)	0.922		
Years of infertility				
Less than 3 years				
3–5 years	−0.82 (-1.85,0.20)	0.115		
More than 5 years	−0.94 (-1.91,0.03)	0.06		
Cycle of the embryo transfer				
1				
2	−0.49 (-1.53,0.54)	0.348	−0.48 (-1.35,0.37)	0.27
3	−1.88 (-3.39,-0.38)	**0.014**	−2.14 (-3.39,-0.88)	**0.001**
3 or more	−2.61 (-4.11,-1.11)	**0.001**	−2.69 (-3.94,-1.43)	**<0.001**
Underlying disease				
None				
Yes	−0.45 (-1.57,0.65)	0.417		

Bold values indicate that *p* < 0.05.

Furthermore, attitude (coefficient = 0.21, 95% CI: 0.11–0.30, *p* < 0.001) and anxiety (coefficient = 0.57, 95% CI: 0.49–0.65, *p* < 0.001) were independently associated with proactive practice (Table 6).

**TABLE 6:** Univariable and multivariable analysis for practice after IVF-ET.

Practice	Univariable analysis		Multivariable analysis	
	coefficient (95%CI)	*p*	coefficient (95%CI)	*p*
			***R*** ^**2**^ =0.4016*	
			**F = 52.41 (*p*<0.001)**	
Knowledge score	0.19 (0.08,0.29)	**<0.001**	0.05 (-0.02,0.14)	0.184
Attitude score	0.56 (0.47,0.65)	**<0.001**	0.21 (0.11,0.30)	**<0.001**
Anxiety score	0.69 (0.62,0.76)	**<0.001**	0.57 (0.49,0.65)	**<0.001**
Age				
20–30 years old				
31–35 years old	0.92 (-0.47,2.31)	0.194	−0.08 (-1.17,1.00)	0.884
36–40 years old	1.47 (-0.09,3.05)	0.066	0.43 (-0.80,1.67)	0.491
≥40 years old	2.20 (0.14,4.27)	**0.036**	1.43 (-0.19,3.06)	0.084
Employment				
Full-time job				
Part-time/Housewife	−0.71 (-2.13,0.70)	0.324		
Freelance	0.26 (-1.09.1.61)	0.701		
BMI, kg/m^2^	0.01 (-0.1,0.16)	0.948		
Living and working environment in the last year				
No pollution				
Yes (noise/decoration/chemical pollution)	−2.23 (-4.04,-0.43)	**0.015**	−0.80 (-2.22,0.60)	0.263
Education				
Primary school and below				
Junior high school	−1.76 (-7.89,4.36)	0.572		
High school/secondary school	−2.36 (-8.45,3.72)	0.445		
College/Undergraduate	−2.64 (-8.61,3.33)	0.386		
Master and above	−1.83 (-7.97,4.29)	0.556		
Monthly per capita income				
<5,000				
5,000–10,000	0.51 (-0.79,1.81)	0.44		
>10,000	1.15 (-0.23,2.54)	0.104		
History of spontaneous abortion				
1				
2 or more	−0.88 (-2.98,1.21)	0.407		
None	0.09 (-1.32,1.51)	0.896		
Reasons for IVF				
Tubal infertility				
Oligospermia, weak or abnormal spermatozoa in the male partner	0.86 (-0.63,2.37)	0.258		
Endometriosis	0.90 (-1.75,3.57)	0.504		
unexplained infertility	0.51 (-1.24,2.27)	0.566		
polycystic ovary syndrome	−0.67 (-2.57,1.22)	0.484		
Ovarian hypoplasia	1.55 (-0.92.4.03)	0.219		
Others	0.85 (-0.92,2.64)	0.345		
Types of infertility				
Primary infertility (never been pregnant before)				
Secondary infertility (previous pregnancy)	−0.41 (-1.48,0.66)	0.454		
Years of infertility				
Less than 3 years				
3–5 years	0.19 (-1.14,1.54)	0.77		
More than 5 years	−0.06 (-1.33,1.21)	0.922		
Cycle of the embryo transfer				
1				
2	−0.69 (-2.05,0.66)	0.317		
3	−1.26 (-3.24,0.70)	0.207		
3 or more	−1.17 (-3.14,0.80)	0.244		
Underlying disease				
None				
Yes	−1.56 (-3.00,-0.12)	**0.033**	−1.10 (-2.22,0.01)	0.053

Bold values indicate that *p* < 0.05.

## Discussion

The results demonstrated that women who underwent IVF-ET had inadequate knowledge and negative attitudes but proactive practices toward ET. Anxiety, income, and reasons for IVF were associated with KAP toward ET. Moreover, knowledge significantly affected attitude, and attitude had a significant positive effect on practice. These findings may provide critical insights into how medical practitioners could develop and implement appropriate interventions and policies to improve KAP levels of ET among women who underwent IVF-ET.

In this study, the average total knowledge score was 19.46 ± 5.06, which was less than 70% of the total knowledge dimension score, suggesting inadequate knowledge regarding ET among the respondents. Previously, knowledge and attitudes of fertility and ART have been investigated among different populations, such as infertile couples/women (Afshani et al., 2016; Rabiei et al., 2022), the general public (Ahmadi and Bamdad, 2017), and university students (Jurkowski et al., 2021). Similar to the present study, most of the respondents in these surveys lacked sufficient information about fertility or ART knowledge. Specifically, the items with low “Very well-known” rates were related to the process of ET, the reason for holding your urine before the transfer, and dietary precautions taken after the transfer. These areas of knowledge deficits should be addressed in future health education or preoperative counseling regarding ET. According to previous reports (Hope and Rombauts, 2010; Daniluk and Koert, 2015; Mori et al., 2021), measures to improve respondents’ knowledge of ET may include online fertility education, educational DVD or brochures, and patient education and care programs.

In terms of the attitude dimension, the average total score of 39.41 ± 5.20 was also below 70% of the total attitude dimension score, indicating the respondents' negative attitude toward ET. Inconsistent with this result, Fauser et al. (2019) demonstrated a positive attitude toward IVF and gamete donation among European men and women. It may be attributed to the different items used to assess attitudes toward ET in the present survey. In addition, differences in the survey populations may be another reason for the conflicting results of the two studies, as only 10% of the people in their studies received IVF treatment. By analyzing the distribution of the attitude dimension, although most respondents recognized the importance of communicating with healthcare professionals about ET treatment and the warm words of healthcare professionals, more than 80% of respondents were still concerned about the success of implantation and delivery of the baby during the IVF-ET procedure. Excessive worry about the outcome of IVF-EMT may lead to adverse psychological emotions, and several studies have shown that some women and families suffer from anxiety, depression, and sleep disorders during IVF-ET treatment. Importantly, the levels of psychophysiological stress before and during treatment may influence the outcome of IVF-ET (Maia Bezerra et al., 2021). Therefore, there is a need for educational and psychosocial interventions to support women and their families physically and psychologically during IVF-ET treatment (Huang et al., 2019).

Different from the knowledge and attitude scores, the respondents showed proactive practice toward ET in the present survey. More than 70% of the respondents rarely or never had sex-related dreams, pain in lower limbs, cold sweats, insomnia, etc., and 30% of the patients had somatic symptoms such as sexual dreams, lower abdominal pain, and insomnia after ET. These findings indicated that the respondents in this survey had low levels of anxiety and active practice after Et. Nevertheless, a survey conducted by Anaman-Torgbor et al. (2021) found that the women undergoing ART were anxious, stressed, exhausted, and financially burdened and that 30% of the women undergoing ART had somatic symptoms such as sexual dreams, lower abdominal pain, and insomnia after Et. This difference may be due to the fact that the time of investigation for their study was when women were seeking ART services, whereas, in the present study, the women were enrolled after ET. In fact, previous studies (Massarotti et al., 2019; Capuzzi et al., 2020) have shown that there is a higher anxiety level among women undergoing infertility treatment before IVF-ET treatment, while women who resorted to IVF could have less perinatal depressive symptoms as the result of a positive outcome of the technique and the satisfaction of the desire to become mothers.

It was noteworthy that these results showed that the respondents’ KAP levels of ET differed by sociodemographic factors. Although some of these influencing factors are non-modifiable (e.g., reasons for performing IVT-ET and years of infertility), other factors may serve as a breakthrough to improve infertile patients’ KAP levels of ET. Specifically, individuals with lower education or income levels had less knowledge about ET, indicating that patients with low income or low education levels are a priority population in need of educational interventions. It does not mean that high-income or high-education patients do not need education, but that they are more likely to already have better knowledge and attitude than the less favored patients. These results are similar to prior research investigating the association between sociodemographic factors and knowledge level of IVF or infertility among women of childbearing age or community residents (Ahmadi and Bamdad, 2017; Arhin et al., 2022). Those results are also supported by the observation that a higher socioeconomic status (which includes education, work, and income, among others, is associated with better health literacy (Svendsen et al., 2020). Interestingly, the practice scores illustrated that lower practice was associated with living or working noise/decoration/chemical pollution environment in the last year and currently suffering from the underlying disease. Nevertheless, the multivariable analysis showed that the independent influencing factors of practice only included attitude score and anxiety score. This may be because, in the univariable analyses, the association between these factors and the level of practice was influenced by other factors and may be a “false association,” which can be easily adjusted and disappear in a multivariable analysis. In combination with the results of correlation analysis and SEM, knowledge significantly affected attitude, and attitude had a significant positive effect on practice and anxiety. Consequently, patient-centered education about ET knowledge may facilitate the development of a positive attitude and a decrease in anxiety for women who underwent IVF-ET.

This study is subject to several limitations that require acknowledgment. Firstly, due to the cross-sectional study design, it is not possible to draw conclusions regarding the effects of influencing factors on improving the KAP levels of respondents. Secondly, the sociodemographic characteristics of participants were not comprehensively included in the analysis, making it difficult to determine the extent to which other factors, such as marital status, may have influenced the responses in this survey. Additionally, it is difficult to generalize the findings of this study to represent all opinions on questionnaire questions, as respondents may have different educational backgrounds, lived in different cities, and had different accents, which may have affected their responses and understanding of questionnaire questions. Finally, the causes of infertility among the participants were mainly tubal infertility or oligozoospermic conditions, but the perception of the male partner with an oligozoospermic condition and its influence on the women’s KAP toward IVF-ET were not analyzed.

In conclusion, women who underwent IVF-ET had inadequate knowledge and negative attitudes but proactive practice toward ET, which were affected by anxiety, income, and reasons for IVF. Moreover, knowledge significantly affected attitude, and attitude had a significant positive effect on practice. Medical professionals should conduct targeted education to improve the awareness of ET-related knowledge among women who underwent IVF-ET to improve the patient’s compliance and enhance their mental health.

## Scope statement

The manuscript titled “Knowledge, Attitude, and Practice of Embryo Transfer among Women who Underwent *In Vitro* Fertilization-Embryo Transfer” investigated the knowledge, attitude, and practice (KAP) of embryo transfer among women who underwent IVF-ET. The study was conducted using a cross-sectional design and surveyed using a self-designed questionnaire from 614 women who underwent IVF-ET between May and November 2023. The results indicated inadequate knowledge and negative attitude but proactive practice toward embryo transfer. The KAP was influenced by anxiety, income, and reasons for IVF. Structural equation modeling and multivariable linear regression analyses revealed significant associations between KAP dimensions and various demographic factors. Specifically, anxiety, income, and reasons for IVF were independently associated with knowledge, while income and number of embryo transfer cycles were associated with attitude. In addition, attitude and anxiety were associated with practice. The study underscores the importance of continuing patient education to enhance their KAP toward embryo transfer. These findings contribute to understanding the factors influencing IVF-ET outcomes and highlight the need for targeted interventions to improve patient education and support.

## Data Availability

The original contributions presented in the study are included in the article/Supplementary Material, further inquiries can be directed to the corresponding author.
