# Supplementary material for: Knowledge, attitude, and practice of embryo transfer among women who underwent in vitro fertilization-embryo transfer
Source: Front Cell Dev Biol. 2024 Aug 7;12:1405250. doi: 10.3389/fcell.2024.1405250 (PMC11335635; doi:10.3389/fcell.2024.1405250)
Supplement: Supplementary file 1 [file Table1.DOCX]

**Supplementary Table 1 Knowledge** **Dimension**

| **Items, n (%)** | **Very well known** | **Somewhat known** | **Not known** |
| --- | --- | --- | --- |
| 1. Do you understand what embryo transfer means? | 285(46.42) | 315(51.3) | 14(2.28) |
| 2. Do you understand the process of embryo transfer? (including whether the transfer is painful, how long it takes, etc.) | 249(40.55) | 334(54.4) | 31(5.05) |
| 3. Do you understand the reason for holding your urine before the transfer? | 271(44.14) | 278(45.28) | 65(10.59) |
| 4. do you think the embryo will fall out if you urinate after the transfer? | 306(49.84) | 236(38.44) | 72(11.73) |
| 5. are you aware of the "ability to work normally after the transfer"? | 315(51.3) | 260(42.35) | 39(6.35) |
| 6. do you know what dietary precautions should be taken after the transfer? | 273(44.46) | 320(52.12) | 21(3.42) |
| 7. do you know how long it takes to take a pregnancy test after the transfer? | 438(71.34) | 167(27.2) | 9(1.47) |
| 8. do you know how soon you can have sex after transplant? | 284(46.25) | 166(27.04) | 164(26.71) |
| 9. Do you know that folic acid supplementation should be started 3 months before pregnancy? | 440(71.66) | 144(23.45) | 30(4.89) |
| 10. during the period of IVF, you can use medicine if you have a cold, but you need to go to a specialist and follow the doctor's prescription. 11. do you know that the embryo transfer can be done after the transfer? | 391(63.68) | 186(30.29) | 37(6.03) |
| 11. Do you know whether you need to stay in bed for a long time after embryo transfer? | 310(50.49) | 264(43) | 40(6.51) |
| 12. After entering the treatment cycle, you need to follow the doctor's instructions to take medication, not to take/stop taking medication on your own, and the importance of regular follow-ups. | 545(88.76) | 66(10.75) | 3(0.49) |
| 13. Do you understand that absolute bed rest is not required after the transfer? | 393(64.01) | 212(34.53) | 9(1.47) |
| 14. Regarding embryo transfer, which treatment process would you most like to know about? |  |  |  |
| a. Before the transfer | 141(22.96) |  |  |
| b. During the transfer | 86(14.01) |  |  |
| c. After the transfer | 387(63.03) |  |  |

**Supplementary Table 2 Attitude Dimension**

| **Items, n (%)** | **Strongly Agree** | **Agree** | **Neutral** | **Disagree** | **Strongly Disagree** |
| --- | --- | --- | --- | --- | --- |
| 1. you think you are able to cope with your fertility problems. | 115(18.73) | 328(53.42) | 146(23.78) | 21(3.42) | 4(0.65) |
| 2. you believe that you are unable to move forward with your life and achieve other life goals and plans because of your fertility problems. | 70(11.4) | 183(29.8) | 185(30.13) | 144(23.45) | 32(5.21) |
| 3. you are concerned about the discomfort you may experience as a result of the embryo transfer procedure. | 61(9.93) | 246(40.07) | 219(35.67) | 77(12.54) | 11(1.79) |
| 4. you are concerned about the success of the implantation and delivery of your baby during the IVF-EMT procedure. | 236(38.44) | 284(46.25) | 81(13.19) | 9(1.47) | 4(0.65) |
| 5. you have feelings of sadness or loss that you will not be able to have children or more children. | 140(22.8) | 245(39.9) | 146(23.78) | 70(11.4) | 13(2.12) |
| 6. you think your family will understand what you are going through. | 182(29.64) | 323(52.61) | 88(14.33) | 18(2.93) | 3(0.49) |
| 7. you are afraid that people will know that you are having an IVF (In Vitro Fertilization - Embryo Transfer). | 73(11.89) | 135(21.99) | 240(39.09) | 135(21.99) | 31(5.05) |
| 8. you think there is a difference between embryo transfer and babies born from natural pregnancies. | 20(3.26) | 80(13.03) | 193(31.43) | 233(37.95) | 88(14.33) |
| 9. you are concerned about the impact of embryo transfer treatment on your daily life and work activities. | 53(8.63) | 194(31.6) | 217(35.34) | 132(21.5) | 18(2.93) |
| 10. you think it is important to cooperate with the doctor's treatment plan and to communicate with the medical staff in a timely manner for the embryo transfer treatment. | 358(58.31) | 224(36.48) | 31(5.05) |  | 1(0.16) |
| 11. you do not trust the surgeon who performed the embryo transfer. | 22(3.58) | 37(6.03) | 122(19.87) | 282(45.93) | 151(24.59) |
| 12. you feel relaxed by the warm words of doctors and nurses. | 357(58.14) | 231(37.62) | 24(3.91) | 1(0.16) | 1(0.16) |

**Supplementary Table 3 Practice Dimension**

| **Items, n (%)** | **Always** | **Often** | **Sometimes** | **Rarely** | **Never** |
| --- | --- | --- | --- | --- | --- |
| 1. I have sex-related dreams after embryo transfer. | 3(0.49) | 5(0.81) | 133(21.66) | 123(20.03) | 350(57) |
| 2. I have pain in my lower limbs after embryo transfer. | 5(0.81) | 20(3.26) | 120(19.54) | 76(12.38) | 393(64.01) |
| 3. I have cold sweats and insomnia after embryo transfer. | 4(0.65) | 21(3.42) | 99(16.12) | 83(13.52) | 407(66.29) |
| 4. I am very worried about the outcome of my pregnancy after embryo transfer. | 91(14.82) | 107(17.43) | 230(37.46) | 138(22.48) | 48(7.82) |
| 5. I feel cold in my lower abdomen and limbs after embryo transfer. | 5(0.81) | 20(3.26) | 102(16.61) | 109(17.75) | 378(61.56) |
| 6. I worry a lot about what to eat after the embryo transfer. | 33(5.37) | 83(13.52) | 195(31.76) | 128(20.85) | 175(28.5) |
| 7. I had cold sweats after the embryo transfer and was frightened. | 6(0.98) | 14(2.28) | 85(13.84) | 81(13.19) | 428(69.71) |
| 8. I was afraid of having an ectopic pregnancy or miscarriage after the embryo transfer. | 59(9.61) | 78(12.7) | 201(32.74) | 164(26.71) | 112(18.24) |
| 9. I am worried that the doctor will transfer the embryos outside the womb. | 13(2.12) | 6(0.98) | 85(13.84) | 54(8.79) | 456(74.27) |
| 10. I felt discomfort, cramps and soreness in my lower limbs after the embryo transfer. | 5(0.81) | 20(3.26) | 85(13.84) | 96(15.64) | 408(66.45) |
| 11. I have lower abdominal pain with bloating after the transfer. | 21(3.42) | 89(14.5) | 218(35.5) | 163(26.55) | 123(20.03) |
| 12. I experienced insomnia after the transfer and woke up easily | 23(3.75) | 67(10.91) | 122(19.87) | 127(20.68) | 275(44.79) |

**Supplementary Table 4. Structural equation model fit**

| Indicators | Reference | Results |
| --- | --- | --- |
| RMSEA | <0.08 Good | 0.546 |
| SRMR | <0.08 Good | 0.106 |
| TLI | >0.8 Good | -1.066 |
| CFI | >0.8 Good | 0.656 |
